# Whole-Genome Resequencing Analysis Reveals Insights into Sex Determination and Gene Loci Associated with Sex Differences in *Procambarus clarkii*

**DOI:** 10.3390/ijms27020938

**Published:** 2026-01-17

**Authors:** Jian Li, Yitian Chen, Yude Wang, Shaojun Liu

**Affiliations:** Engineering Research Center of Polyploid Fish Reproduction and Breeding of the State Education Ministry, Hunan Normal University, Changsha 410081, China

**Keywords:** *Procambarus clarkii*, resequencing, sex determination, sex chromosome, sex-marker

## Abstract

Since the molecular mechanisms underlying sex determination in *Procambarus clarkii* are still unclear, it is important to investigate the genetic basis of sex determination in crustaceans. Currently, the molecular mechanisms of sex determination and the gender-specific markers in this species remain poorly understood. In this study, a total of 14,046,984 SNPs and 2,160,652 InDels were identified through genome-wide resequencing of 89 individuals (45 females and 44 males). Further analysis confirmed that the candidate chromosome was Chr38, the sex determination system was identified as XY, and the sex determination region was located at Chr38: 6,000,000–21,100,000 bp. A pair of sex-specific molecular markers has been identified based on a 21 bp female-specific insertion within the candidate sex-determining region. Additionally, SOAT, NPC1, PTGS2, FANCD1, and VAlRS were identified as candidate sex-determining genes through the screening of candidate genes and RT-qPCR validation analysis. These findings provide a robust foundation for investigating sex-determining mechanisms in crustaceans. Through the integration of genome-wide association studies (GWAS), selection signals, and transcriptome analysis, we identified, for the first time, genes associated with sex determination, growth, and immunity. These genes represent promising candidates for further functional studies and genetic improvement in *Procambarus clarkii*.

## 1. Introduction

Sex determination (SD) refers to the mechanism that determines whether a zygote develops into a male or a female [1]. Crustaceans exhibit diverse sexual systems, including hermaphroditism, environmental sex determination (ESD), and genotypic sex determination (GSD). GSD can be further classified into XY and ZW systems based on whether males and females are heterogametic or homogametic. XY systems are present in many animals, including humans, Drosophila, and Caenorhabditis elegans, where the Y chromosome is significantly degraded or deleted [2]. In contrast, ZW systems are characterized by females being heterogametic (ZW) and males being homogametic (ZZ). The ZW sex determination system is observed in birds [3], reptiles [4], insects [2], and certain crustaceans [5].

Sexual development includes two main processes: sex determination and sex differentiation. Sex determination refers to the development of undifferentiated gonads into testes or ovaries, while sex differentiation involves the induction of phenotypic sex traits following gonad development. In general, the sex-determining systems in animals and plants are either XX/XY or ZZ/ZW [6]. For instance, *Macrobrachium rosenbergii* has a clearly defined ZZ/ZW sex-determining system, and sex-specific markers have been developed to facilitate sex inversion [7,8]. In a study on the black tiger shrimp [9], researchers identified male-specific SNP markers, which support the proposed XX/XY sex determination system [10]. Genome-wide resequencing enables rapid and accurate identification of genetic differences between males and females, making it easier to develop sex-specific molecular markers. Genome-specific molecular markers have been developed through whole-genome resequencing include *Megalobrama amblycephala* [11], *Spinibarbus hollandi* [12], and *Leiocassis longirostris Günther* [13]. The development of markers based on single nucleotide polymorphisms (SNPs) and insertions and deletions (InDels) reduces the chances of failure in trait-associated marker development and provides new insights into sex-determining [14,15,16,17].

*Procambarus clarkii*, which entered China in the 1930s, has become a significant economic aquatic organism due to its delicious meat and high nutritional value [18]. Among commercially farmed species, global production of this species reached 2.47 million tons, representing 22% of all crustacean output in 2020 [19]. In 2024, the output of *Procambarus clarkii* in China reached 3.44 million tons, which accounted for 9.75% of the total freshwater aquaculture output and 60.44% of the crustacean output. This represented an increase of 9.07% compared to 2023 [20], establishing it as a key component of China’s aquaculture economy. Additionally, the rapid growth of the *Procambarus clarkii* industry has contributed to advancements in aquaculture technology. *Procambarus clarkii* has emerged as one of the most economically important aquatic species in China [21]. They exhibit sexual dimorphism, with higher meat percentage compared to males of the same weight [22]. Significant differences in size and growth rate between males and females have been observed in many aquatic species [23]. For instance, female white shrimp grow significantly faster than males [24]. Culturing all-female *Procambarus clarkii* can greatly improve aquaculture efficiency. However, effective sex control requires a thorough understanding of the sex-determining system and the development of sex-specific molecular markers. Although sex chromosomes have been discovered in crustaceans [25], most have not yet been found to have heteromorphic chromosomes. So far, no heteromorphic chromosomes have been found in the species of *Procambarus clarkii*. In depth studies have been conducted on the chromosomes of the *Procambarus clarkii*, but no sex chromosomes have been found. Studies have shown that the chromosome count is 2n = 188 [26]. The genome size obtained through chromosome-level genome assembly is 4.03 Gb [27]. Despite its economic importance, the sex-determining system of *Procambarus clarkii* remains poorly understood, underscoring the need for further research.

This study aims to investigate the sex determination system of *Procambarus clarkii* through whole-genome resequencing. We concentrated on identifying key genes associated with immune-related and growth differences between males and females, developing sex-specific molecular markers, and establishing a foundation for understanding the molecular mechanisms of sex determination in *Procambarus clarkii*. Additionally, our findings provide valuable references for exploring sex determination systems in other crustacean species.

## 2. Results

### 2.1. Resequencing Data and SNP Calling

**Figure 1:**
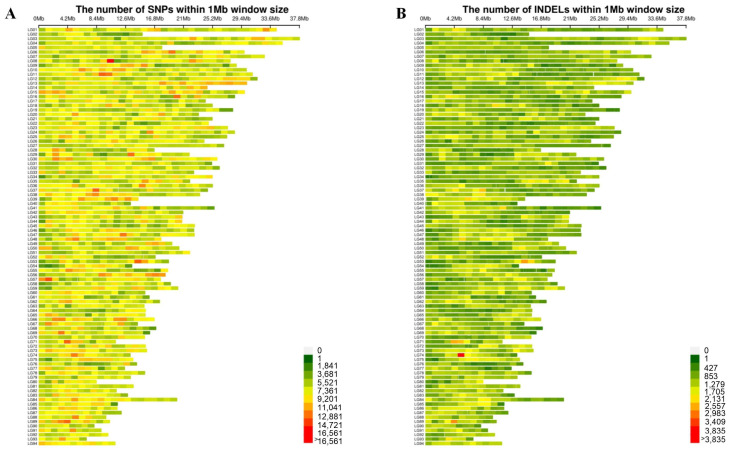
The density and distribution of genotyped SNPs and InDels on each chromosome. (**A**) SNP density and distribution on each chromosome. (**B**) InDel density and distribution on each chromosome.

Whole-genome resequencing was performed on male and female populations. Raw data were filtered and quality assessed prior to information analysis. Using FASTP, low-quality data were removed, resulting in a total of 2644.85 Gb of data across all samples, with an average of 29.72 Gb per sample. The average Q30 value for clean reads across all samples was 96.08%, and the high-quality sequencing results are obtained when the proportion of data GC was below 44% (Table 1, Table S1 in Supplementary Material S1). The mapping rate of clean data to the reference genomes by BWA is 90.32% to 99.01% (Table 2, Table S2 in Supplementary Material S2). After alignment, the average coverage depth of 10.86× per individual. SNP identification was performed using GATK based on alignment results. Further filtering of extracted SNPs was conducted to improve SNP quality for subsequent GWAS analysis. After filtering, a total of 14,046,984 SNPs and 2,160,652 InDels were identified. Density map of SNPs and InDels reveal their distribution across each chromosome (Figure 1).

### 2.2. Comparison of LD

**Figure 2:**
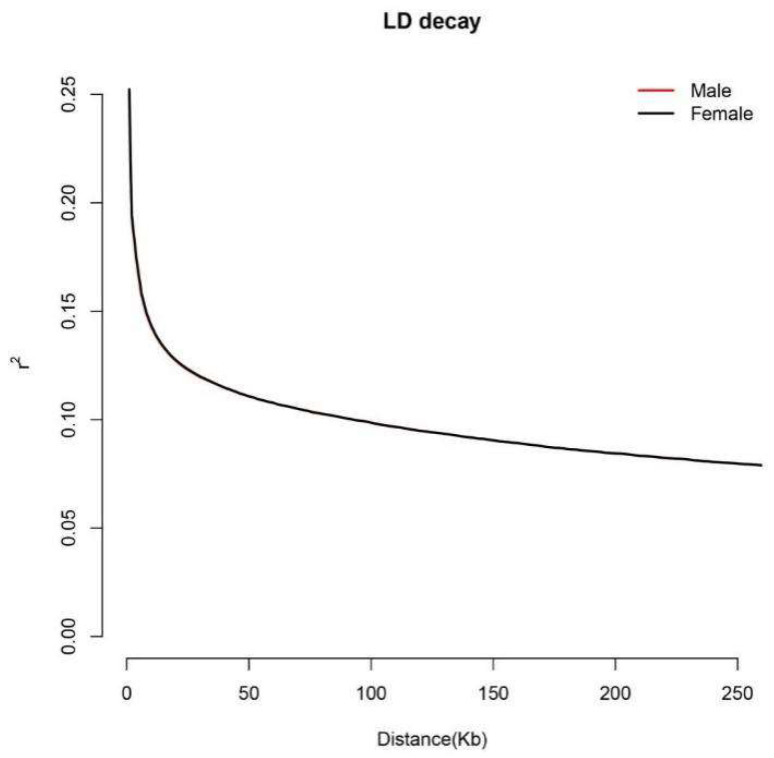
Comparisons of LD among male and female populations.

For 14,046,984 SNP loci across 89 samples, the linkage disequilibrium (LD) decay between male and female molecular markers in the *Procambarus clarkii* population was calculated using PopLDdecay software(v3.41). The population’s LD level was quantified using r^2^. The results indicated no significant difference in LD decay rates between male and female populations (Figure 2).

### 2.3. Identification and Mapping of Sex-Linked Variant

**Figure 3:**
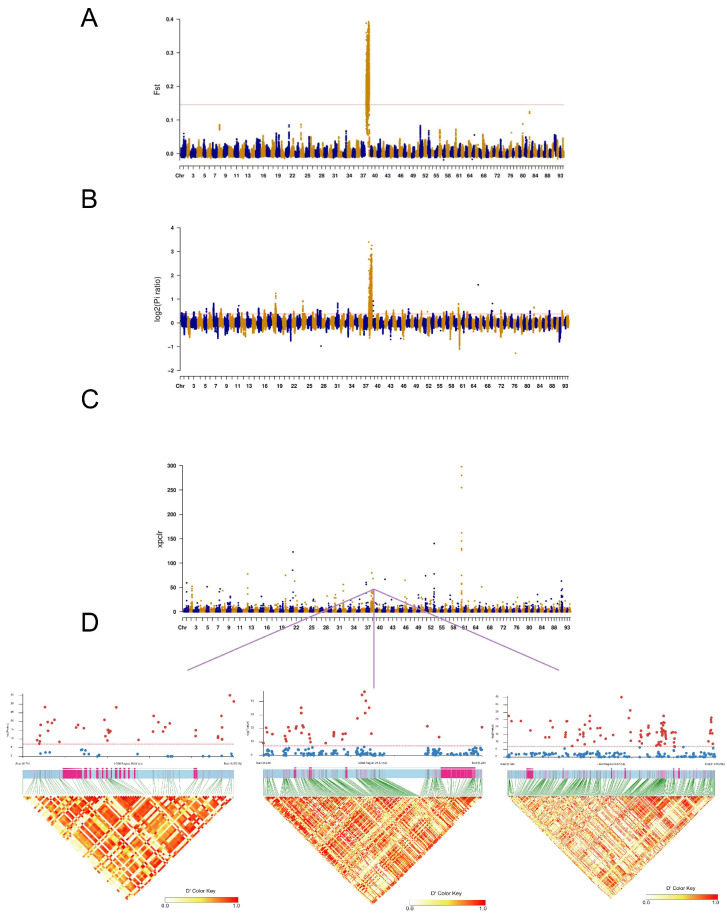
Identification of selective signals based on comparing male and female populations and Linkage Disequilibrium (LD) analysis of Chromosomal positions of candidate sex determination genes. (**A**) Manhattan plot of signal selection using the Fst methods. (**B**) Manhattan plot of signal selection using the log_2_(Pi ratio) methods. (**C**) Manhattan plot of signal selection using the XP-CLR methods. Dashed lines represent the threshold of the top 5% values. The area above this line is regarded as a potential candidate region that may contain the selected feature. (**D**) LD analysis spanning the physical position of chromosome 38. The top and bottom shows the *p*-values of SNP in the genomic region of candidate sex-determining genes and the LD heatmap.

In population genetics, the fixation index FST is commonly used to measure the degree of populations differentiation. An FST value greater than 0.15 indicates significant genetic differentiation between the two populations. Thus, higher FST values correspond to greater genetic differentiation and more pronounced differences between populations. FST values between female and male groups were calculated using vcftools software v4.0, and the results were plotted across the entire genome (Figure 3A). The results revealed FST values exceeding 0.25 on chromosome 38. Additionally, Pi analysis was conducted to estimate expected heterozygosity at each locus, calculated as the average of sequence differences across samples. The results are shown in Figure 3B. XP-CLR focused on multilocus allele frequency differences between the two populations, employing deterministic models to selective sweep on single nucleotide polymorphisms (SNPs), as illustrated in Figure 3C. Chromosome 38 was identified as the candidate chromosome for *Procambarus clarkii* through FST, Pi, and XP-CLR analyses. Moreover, the region Chr38: 6,000,000–21,100,000 bp was designated as a candidate region for sex determination. Through FST, Pi, and XP-CLR analyses, 189, 237, and 1523 genes were identified, respectively. The most significant selective sweep was detected on chromosome 38. Based on the results of FST, Pi, and XP-CLR analyses, 151 common signal selection windows within the top 1% were identified, overlapping 134 genes, and all were located on chromosome 38.

### 2.4. Selection Signatures Between Male and Female Populations

A total of 189, 237, and 1523 genes were identified in male and female populations of *Procambarus clarkii* using FST, Pi, and XP-CLR methods, respectively. Cross-analysis of these three methods identified 134 candidate genes (Figure 3). The major sex-determining candidate genes (SOAT, NPC1, PTGS2) identified through selective sweep. We conducted an linkage disequilibrium (LD) analysis to study the regions of LD between the sex-determining gene loci, in order to identify SNPs located within the genomic regions of the three candidate genes. These genes are located on Chr38: 6,000,000–21,100,000, and the linkage disequilibrium of SNP loci associated with these genes is illustrated (Figure 3D). We observed that most SNPs in this region exhibited high linkage disequilibrium, with FST values reaching 0.25. To identify genes associated with growth and immune traits, GO enrichment analysis elucidated the functional roles of these candidate genes. The analysis identified CHST11, ANILLIN, ERAD, SPO, cytokine receptor, and CES4A as key genes associated with growth-promoting factors. The enriched GO terms included “developmental growth” (GO:0048589), “actin binding” (GO:0003779), “cell division” (GO:0051301), “cell migration” (GO:0016477), “skeletal system development” (GO:0001501), “eye development” (GO:0001654), “angiogenesis” (GO:0001525), and “positive regulation of cell population proliferation”(GO:0008284), all of which were associated with growth and development. ApoD and ERAD were associated with metabolism-related processes, including “glucose metabolic process”(GO:0006006), “lipid metabolic process” (GO:0006629), and “ubiquitin-dependent protein catabolic process” (GO:0006511). Potential genes associated with immune traits were also identified, such as WNT-2B, VPS13A, WDR92, RS1, and FABP1. Enrichment analysis showed that these genes were enriched in multiple pathways, including the “Wnt signaling pathway” (GO:0016055), “lipid transport” (GO:0006869), “ATP binding” (GO:0005524), “apoptotic process” (GO:0006915), and “lipid binding” (GO:0008289).

### 2.5. Genome-Wide Association Studies

**Figure 4:**
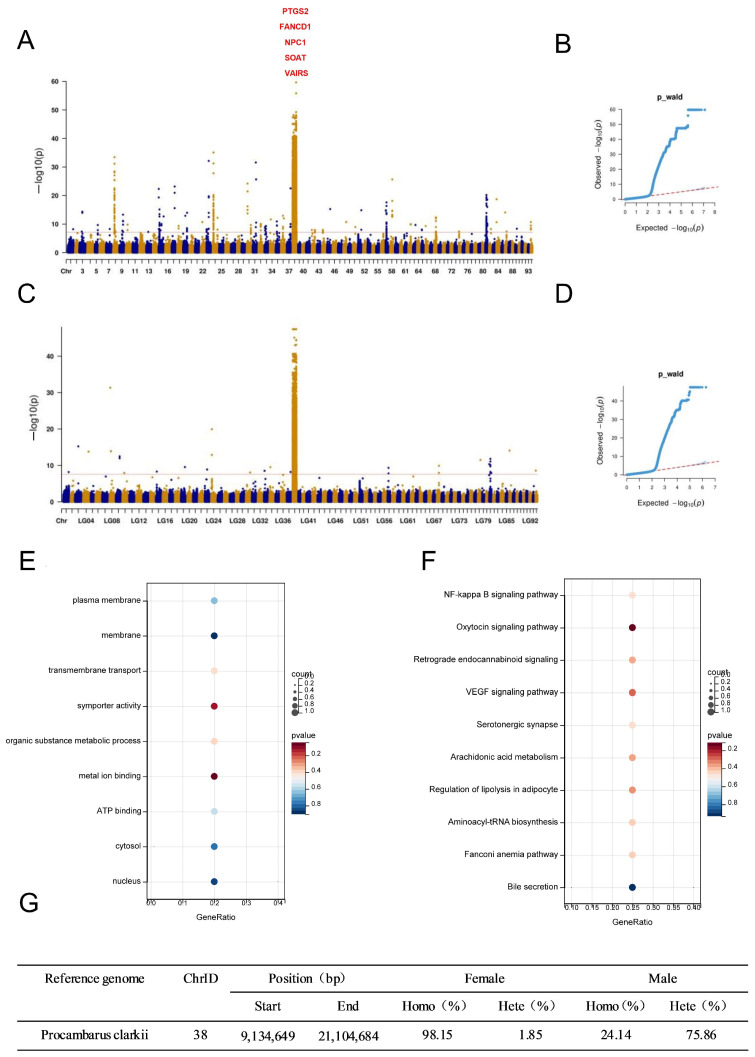
Genome-wide association analysis was conducted on male and female populations using SNPs and InDels data. (**A**,**B**) The Manhattan diagram. (**C**,**D**) The quantile–quantile (Q–Q) diagram. (**E**) Gene Ontology functional enrichment bubble plot of candidate sex determination genes. (**F**) An overview of the KEGG pathways enriched in candidate sex determination genes. (**G**) Statistical analysis of heterozygosity levels of SNPs in sex-determining regions.

The genetic mechanism of the sex determination (SD) region in *Procambarus clarkii* remains poorly understood. A genome-wide association study (GWAS) of 44 females and 45 males of *Procambarus clarkii* revealed a significant peak on chromosome 38, which was distinct from other chromosomes. Statistical analysis of all SNP loci in the candidate sex-determining region revealed that 98.15% of significant SNPs were homozygous in females, compared to only 21.14% in males. This indicates a higher homozygosity rate in females compared to males, supporting previous research suggesting that the sex determination system in *Procambarus clarkii* is consistent with the XY system (Figure 4G)). Genome-wide association analysis (GWAS) was conducted on 14,046,984 SNPs obtained from male and female populations of *Procambarus clarkii* using GEMMA software. The genetic relationship matrix among samples was calculated, and the obtained matrix was used as a covariate in the association analysis. In GWAS, Manhattan plots and quantile–quantile (Q–Q) plots were used to visualize the results (Figure 4). We utilized genome-wide resequencing data from 89 male and female individuals for GWAS to examine all mutation loci, which led to the identification of 151 candidate genes. The major sex-determining candidate genes (SOAT, NPC1, PTGS2), previously identified through selective sweep, overlapped with GWAS signals, highlighting the importance of these genomic regions in shaping characteristics. Furthermore, we identified two new sex-determining candidate genes (FANCD1, VAlRS). FANCD serves as a potential sex biomarker, while VAlRS plays a crucial role in spermatogenesis. The five candidate genes were analyzed through selective sweep analysis and genome-wide association studies (GWAS).

### 2.6. Functional Annotation of Candidate Genes by GO and KEGG Pathway Analysis

To further explore the major biochemical and candidate genes involved in signaling pathways, we conducted GO and KEGG pathway enrichment analyses using public databases. Following a comparison with the NR protein database, the GO and KEGG functions of the selected genes were refined. As shown in Figure 4E, five distinct genes (SOAT, NPC1, PTGS2, FANCD1, VAlRS) were represented in various GO terms (Table S3, Supplementary Material S3). We performed KEGG enrichment analysis, and all significant pathways were visualized in a bubble chart (Figure 4F). Notably, only four genes (SOAT, PTGS2, FANCD1, VAlRS) were enriched in different KEGG pathways (Table S4, Supplementary Material S4).

### 2.7. Major Candidate Genes Associated with Growth and Developmental Traits

Annotation of significant GWAS signals revealed associations related to growth and development, and identified several novel genes. The most significant SNP was situated on chromosome 38. Differences in growth rate and size between male and female chelae have been a central focus of modern breeding programs. To investigate differences in growth and development between female and male *Procambarus clarkii*, we conducted a GWAS five candidate genes (DLP, GFI-1B, MLP 84B, SMYD, and IDE) were identified through our screening process. Previous reports indicate that the differential expression of DLP in various cells leads to variations in cell growth and apoptosis. GFI-1B, an erythrocyte regulatory factor, is known to play a specific role in regulating erythrocyte growth. MLP 84B is a cytoskeletal protein specifically expressed in striated muscle; mutations in MLP 84B have been shown to impair muscle tissue function in animals. The SMYD protein is widely recognized as an epigenetic regulator of myogenesis and cardiomyocyte differentiation during early development, with abundant expression in both myocardium and skeletal muscle. IDE, a target protein of Sca-1 (stem cell antigen-1), a marker gene for myogenic progenitor cells, maintains myoblast proliferation in a differentiated state under siRNA interference, leading to a slowdown in myoblast differentiation. These genes are likely to play a crucial role in regulating growth and body size in *Procambarus clarkii*.

### 2.8. Major Candidate Genes Associated with Immunity

To investigate immune differences between female and male shrimp during breeding, we conducted a GWAS to identify immune-related genes in *Procambarus clarkii*, screening four candidate genes: JAGN, DPP1, CHST11, and CCT8. JAGN is known to play a crucial role in humoral immunity and antibody glycosylation in both mice and humans. DPP1 is a member of the papain-like cysteine protease family. The DPP1 gene was successfully cloned and expressed in Escherichia coli BL21, eliciting a robust immune response. CHST11 is involved in regulating pathways associated with tumor growth, metastasis, and immune response. Overexpression of CCT8 inhibit the activated B cells, eosinophils, and natural killer cells, suggesting their involvement in immune mechanisms. Overall, we propose that these candidate genes may provide valuable insights into the differences in immunity and survival between male and female.

### 2.9. Development and Verification of Sex-Specific Markers

**Figure 5:**
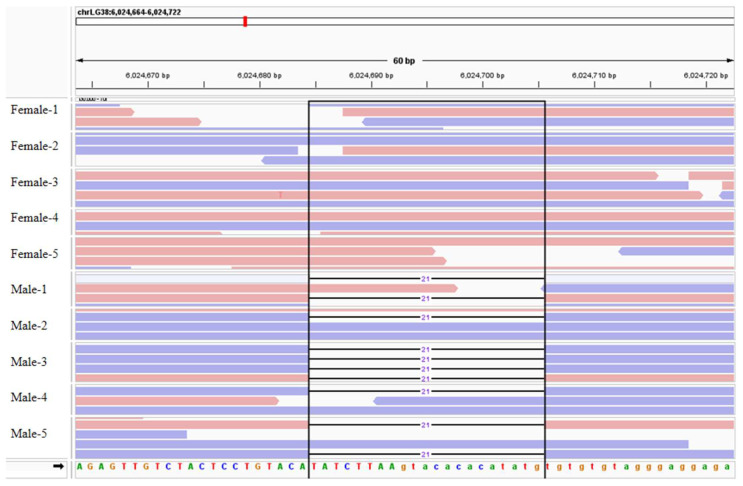
Comparative alignment of the sequencing reads from both sexes. The location within the black line frame represents a Female-specific insertion fragment (21 bp), which is situated at Chr38: 6,024,689 and 6,024,710 bp.

**Figure 6:**
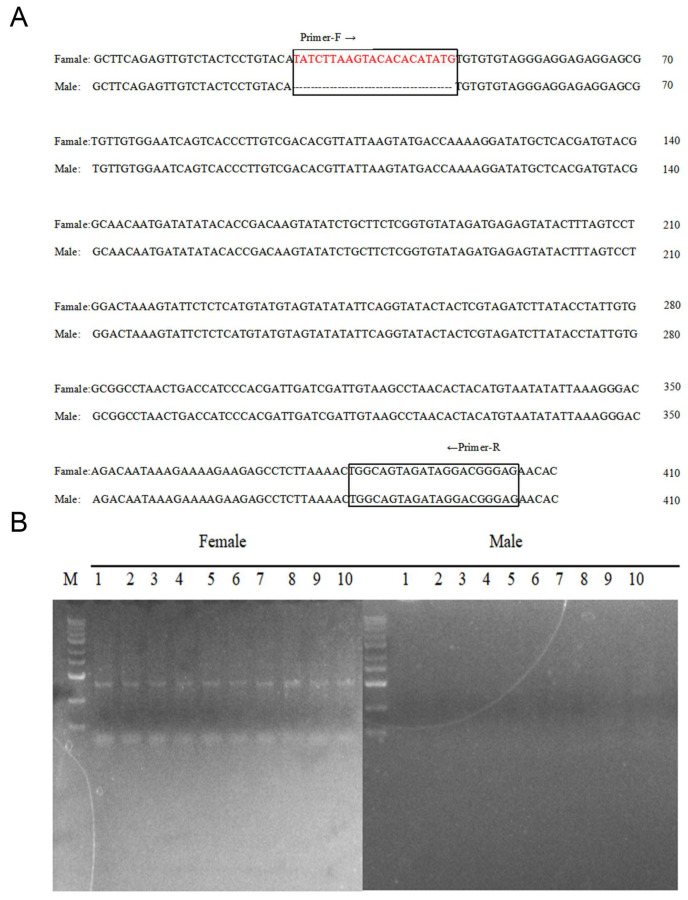
Primer amplification sequences and their specific molecular marker validation. (**A**) Male-specific sequence and primer design, with red font indicating the 21 bp female-specific insertion sequence, and the black box represents the primer design sequence. (**B**) Application of the PCR-based method for genetic sex identification of *Procambarus clarkii* (female and male) population.

Candidate chromosomes and sex-determining regions were manually examined for candidate loci using IGV software v.2.16.2. As shown in Figure 5, a 21 bp Indel sequence (TATCTTAAGTACACATATG) was identified in all females, located between 6,024,689 and 6,024,710 bp on Chr38. We used faidx function of samtools to extract sequences near the Indel, with the range being Chr38: 6,024,664–6,025,164. Primers were designed using Primer Premier v6.0 software. We designed a pair of primers (Forward primer: ATATCTTAAGTACACATATG; Reverse primer: CTCCCGTCCTATCTACTGCCA) (Figure 6). PCR validation was conducted using these primers. As shown in Figure 6A,B, PCR results demonstrated that all female individuals observed a single band, whereas male individuals did not. These findings indicate that primers identified through these primers can be effectively used for rapid gene sex identification in *Procambarus clarkii*.

### 2.10. Expression of Candidate Genes in the Gonads

**Figure 7:**
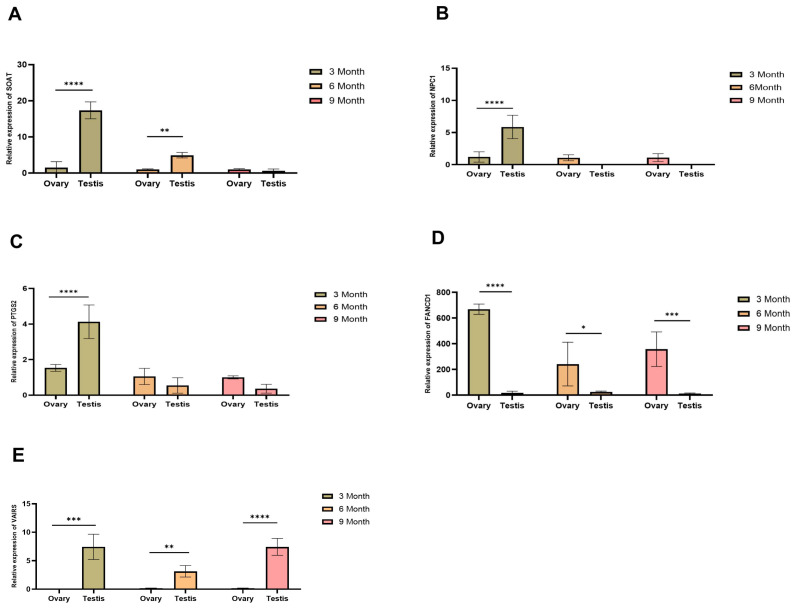
The expression of selected candidate sex determination genes SOAT (**A**), NPC1 (**B**), PTGS2 (**C**), FANCD1 (**D**), and VAlRS (**E**). Verified by RT-qPCR. The *y*-axis represents the expression levels of these genes. The asterisk indicates statistical significance (*  *p* < 0.05, **  *p* < 0.01, ***  *p* < 0.001, **** *p* < 0.0001).

Using RT-qPCR (real-time quantitative PCR) to validate the differential expression of these sex candidate genes in gonadal tissues. The results indicated that the expression levels of four genes (SOAT, NPC1, PTGS2, VAlRS) were consistently higher in testis tissue at 3 months of age compared to ovary tissue (*p* < 0.001) (Figure 7A–C,E). Additionally, FANCD1 gene expression levels were significantly higher in ovary tissue at different stages compared to testis tissue (Figure 7D). At 3 months of age, SOAT and VAlRS expression levels were consistently higher in testis tissue (*p* < 0.001). At 6 and 9 months of age, no significant differences were observed in the expression levels of NPC1 and PTGS2 in gonad tissues. However, at 6 months, SOAT expression was significantly higher in testis tissue (*p* < 0.01), whereas at 9 months, no significant difference was detected in SOAT expression between testis and ovary tissues. We speculated that the differential expression in gonadal tissues may be related to the mechanisms of sex determination.

### 2.11. Transcriptome Analysis

**Figure 8:**
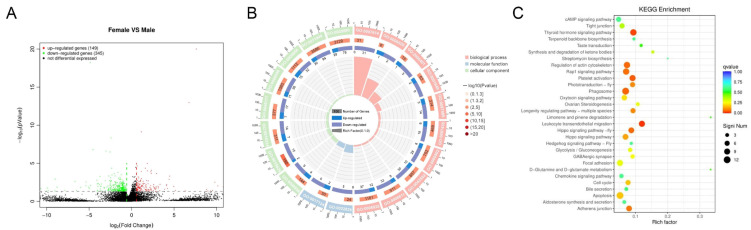
(**A**) The volcano plot shows common and specific DEGs; red dots represent up-regulated genes, and green dots represent down-regulated genes. (**B**) GO enrichment circle diagram of cyan module; (**C**) The KEGG enrichment bubble plot of volcano DEGs shows the top 30 pathways of the DEGs enriched in the KEGG analysis.

**Figure 9:**
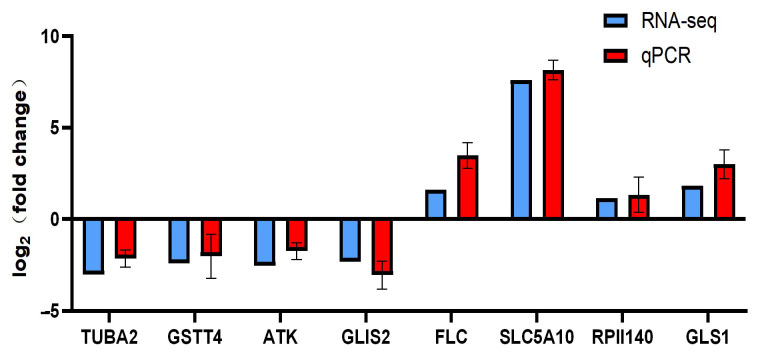
Verification of gene expression patterns of RNA-seq by qRT-PCR in the muscle of *procambarus clarkii*.

A total of 494 genes were identified as differentially expressed between female and male individuals, with 149 up-regulated and 345 down-regulated. GO enrichment analysis revealed that the differentially expressed genes were significantly enriched in the cyan modules (Figure 8A). GO enrichment analysis indicated that differentially expressed genes in the blue module were primarily associated with biological processes such as osteoblast differentiation and cell morphogenesis, molecular functions including wide pore channel activity, transmembrane transporter activity, and SH3 domain binding, as well as cellular components like the axonal dynein complex and extrinsic component of membrane (Figure 8B). Among the top 30 most enriched KEGG pathways, apoptosis and phagosome pathways were the most significantly enriched, with 12 differentially expressed genes (DEGs). The regulation of the actin cytoskeleton, platelet activation, and Rap1 signaling pathway followed, each containing 10 DEGs. Additionally, several growth and immune-related pathways were identified, including the TGF-beta signaling pathway, Toll and Imd signaling pathway, and thyroid hormone signaling pathway (Figure 8C). RT-qPCR analysis was conducted to verify transcriptome results (Figure 9). The results confirmed that RNA-seq data are accurate.

These findings indicated that there are differences in growth and immune between male and female *Procambarus clarkii*, which are also observed in practical breeding process. These results provided theoretical support for applications in breeding and genetic improvement.

## 3. Discussion

Different aquatic organisms exhibit diverse sex chromosome determination mechanisms, such as the XY and ZW systems, which involve complex regulatory networks. For example, *Macrobrachium rosenbergii* has a ZW sex chromosome system that is uniquely determined by a single gene [28]. *Procambarus clarkii* possess a larger number of chromosomes compared to most other species, making it challenging to distinguish autosomes from sex chromosomes using conventional methods. The gonad plays a crucial role in development, yet gene regulatory mechanisms govern remain poorly understood. Therefore, we select male and female *Procambarus clarkii* populations for whole-genome resequencing. This study aims to deepen our understanding of the regulatory mechanisms underlying male and female sex development and provide novel insights into the study of sex development in *Procambarus clarkii*.

*Procambarus clarkii*, a crustacean, has emerged as one of the most economically important aquatic species in China. Among them, notable differences in growth rates between males and females have been observed, such as in *Macrobrachium orientale* and *Macrobrachium nipponense*, where males grow faster than females [29,30]. *Procambarus clarkii* exhibits significant sexual dimorphism [31]. Under identical weight conditions, female *Procambarus clarkii* demonstrate a higher muscle occupancy rate compared to males [22]. Therefore, investigating sex determination, developing sex-specific molecular markers, and understanding the influence of sex on growth and development are essential. These efforts aim to enable single-sex populations through sex control breeding technology, thereby enhancing the growth rate and economic value of *Procambarus clarkii* in breeding. The development of sex-specific molecular markers represents a crucial technological approach for achieving sex control and single-sex population breeding.

High-throughput sequencing technologies have enabled the successful development of numerous sex-specific molecular markers in various aquatic organisms. Whole-genome resequencing is a key technique in animal and plant molecular breeding, particularly for detecting large sex-linked regions. It is highly effective in identifying gender differences between male and female genomes and has become an important method for identifying gender markers, such as in *Megalobrama amblycephala* [11] and *Spinibarbus hollandi* [12]. These results demonstrate that whole-genome resequencing for genotyping is both efficient and accurate in identifying sex-specific markers. In this study, we used genome-wide resequencing to identify valid and reliable sex-specific markers in *Procambarus clarkii*. A pair of sex-specific primer was designed within a 21 bp sequence that is unique to females and this male-specific sequence can amplify a single specific band in male individuals. Furthermore, through comparative analysis and annotation of the *Procambarus clarkii* reference genome, we identified inserted female-specific 21 bp sequences in noncoding regions. However, whether the female-specific insertion of the 21 bp sequence affects the expression of upstream and downstream genes in the female genome remains to be further investigated.

Male and female *Procambarus clarkii* exhibit differences in growth rate, disease resistance, and other economically important traits crucial for crustacean aquaculture and economic efficiency. Determining the genetic sex of individual *Procambarus clarkii* remains a significant challenge, hindering the development of sex-controlled breeding and the sex-determination mechanism of this species remains unclear. In this study, we conducted genome-wide resequencing and genome-wide association studies (GWAS) on 89 *Procambarus clarkii* individuals, comprising both females and males. We identified a total of 14,046,984 SNPs and 2,160,652 Indels in this study (Figure 1). We identified candidate sex chromosomes and sex-determining regions of *Procambarus clarkii* using three methods: FST, Pi, and XP-CLR. The candidate sex chromosome of *Procambarus clarkii* is Chr38, with the sex-determining region spanning 6,000,000 to 21,100,000 bp on Chr38. Using candidate sex-determining regions, along with the analysis of identified sex-dimorphic SNPs and Indel, we screened a large number of sex-dimorphic variants, including SNPs and Indels. Finally, we discovered a female-specific 21 bp insertion sequence located between 6,024,689 and 6,024,710 bp on Chr38 (Figure 5). Based on this characteristic, we designed a pair of specific primers that successfully amplified bands exclusively in females and not in males (Figure 6). Through resequencing technology, we developed a pair of sex-specific molecular markers for *Procambarus clarkii*, creating an efficient tool for sex-controlled breeding. This advancement is significant for improving economic yields in aquaculture.

Several genes associated with sex selection were identified using methods based on three selective sweep analyses: FST, Pi, and XP-CLR. We identified three candidate genes in the sex-determination candidate regions (SOAT, NPC1, PTGS2), suggesting their potential involvement in sex determination. GO and KEGG enrichment analyses provide insights into gene functions and roles, as well as their corresponding biological processes, pathways, and diseases. These tools are essential for interpreting biological data and guiding future research. In this study, we performed GO and KEGG functional enrichment analyses on candidate genes. Five genes (SOAT, NPC1, PTGS2, FANCD1, VAlRS) were observed to be enriched in various GO terms, and four genes (SOAT, PTGS2, FANCD1, VAlRS) were enriched in different KEGG pathways. Through selective sweep of three candidate genes and GWAS signal overlap, we identified two new sex-determining candidate genes (FANCD1, VAlRS) using GWAS. Five candidate sex-determining genes were identified through genome-wide resequencing, which are essential for understanding sex determination in crustaceans and offer valuable insights for future practical applications.

We found that SOAT expression levels in testis and ovary tissues were significantly higher at 3 and 6 months of age compared to ovary tissues at other stages of *Procambarus clarkii* (Figure 7A). Studies have shown that SOAT gene expression in human and mouse testicular tissue reaches its highest level [32,33]. Due to its high expression in spermatogenic cells, including primary spermatocytes, secondary spermatocytes, and sperm cells, SOAT is thought to play a crucial role in spermatogenesis [34]. NPC1, an endogenous cholesterol transporter, is highly conserved and widely distributed across vertebrates and invertebrates [35]. Studies in silkworm have shown that NPC1 is highly expressed in testis and ovary tissues. Additionally, some studies have demonstrated that NPC1a played a key role in the sperm formation process of Drosophila [36,37]. However, few studies have been conducted in crustaceans. In *Macrobrachium nipponense*, the expression level of NPC1 is positively correlated with ovarian maturity, playing a key role in ovarian development [38]. In this study, the expression level of NPC1 in gonads of *Procambarus clarkii* was significantly higher in testis tissue compared to ovary tissue at 3 months of age, but no significant difference was observed between the ages of 6 and 9 months (Figure 7B). PTGS2, also known as prostaglandin endoperoxide synthase-2, is thought to be involved in spermatogenesis and steroidogenesis [39]. Two key somatic cell types of the testis, Leydig and Sertoli cells, express the inducible isoenzyme PTGS2 and produce PGs, suggesting that PGs may play an important role in testicular development, steroidogenesis, and spermatogenesis in various species [40,41]. In this study, we found that PTGS2 expression was significantly higher in the 3-month-old testis compared to the ovary (Figure 7C). Research on the FANC gene family in teleost fish is still limited. Studies of the FANC gene family in teleost fish have been limited to zebrafish, focusing on their roles in development and sex determination [42]. Interestingly, mutations in at least two of these genes (fancl and fancd1) have been shown to result in female-to-male sex reversal. The transcript of fancd1 (brca2) localizes to the animal pole of zebrafish oocytes and is crucial for maintaining normal oocyte nuclear architecture. The data indicate that the fancd2 gene as a sex biomarker and binds to its respective miRNA for regulation [43]. In this study, we found that FANCD1 expression was significantly higher in ovary tissue compared to testis tissue of *Procambarus clarkii* at 3, 6, and 9 months of age (Figure 7D). VAlRS-knockdown in Drosophila testes resulted in male sterility and early defects in spermatogenesis. Additionally, VAlRS-knockdown testes exhibited severe defects in the transition from spermatocyte to primary spermatocyte. Together, these findings suggest that VAlRS played a crucial role in spermatogenesis [44]. In this study, we found that VAlRS expression was significantly higher in testis tissue compared to ovary tissue of *Procambarus clarkii* at 3, 6, and 9 months of age (Figure 7E). Based on our findings, we propose that the differential expression of sex candidate genes identified through genome-wide resequencing may be associated with the underlying mechanism of sex determination. Our results not only further clarify the candidate genes for sex determination in *Procambarus clarkii* but also provide valuable theoretical insights for crustacean sex research.

In this study, we used whole-genome resequencing to identify candidate sex-determining genes and screen genes exhibiting differences in growth rate and immunity response between male and female individuals, aiming to further investigate sex determination and variation differences in *Procambarus clarkii*. Further investigation is required to elucidate the mechanisms of these genes in the sex determination process of *Procambarus clarkii*. In conclusion, this study provides important insights into the mechanisms of sex determination in *Procambarus clarkii*, as well as the development of sex-specific molecular markers and the application of genome-wide resequencing analysis for identifying candidate sex-determining genes. These findings provide further insights into sex-determining mechanisms in *Procambarus clarkii* and other crustaceans. These findings have significant implications for sex determination mechanisms in *Procambarus clarkii*, sex-controlled breeding, and other aquaculture-related fields.

## 4. Materials and Methods

### 4.1. Ethics Statement

The animal care and experimental protocols were certified by a professional training course for laboratory animal practitioners held by the Institute of Experimental Animals, Hunan Province, China. Add 2 L of water to a 5 L glass container, then weigh 200 mg of MS-222 solid powder and dissolve in water. After the powder was dissolved, the experiment *P. clarkii* was put into the water. The experimental *Procambarus clarkii* samples were deeply anesthetized before collecting gonadal and muscle tissues.

### 4.2. Sample Collection and Whole Genome Re-Sequencing

The 45 female and 44 male *Procambarus clarkii* were obtained from the State Key Laboratory of Developmental Biology of Freshwater Fish, Hunan Normal University, China. The sex of each individual was determined through anatomical examination of the gonad. DNA was extracted from muscle tissue of *Procambarus clarkii* using the Genome Extraction Kit (Qingdao, China) following the manufacturer’s instructions. The quality and integrity of the DNA were assessed using a NanoDrop spectrophotometer (Thermo Fisher Scientific, Waltham, MA, USA) and 1% agarose gel electrophoresis. Sequencing was conducted on the NovaSeq 6000 platform (Illumina, San Diego, CA, USA) using a 150 bp paired-end. The raw reads were quality-filtered using fastp v0.23.4, yielding clean data. The clean reads were aligned to the *Procambarus clarkii* reference genome using BWA-MEM [45] to obtain the gVCF for each sample. Joint calling was performed using GATK (v4.0) [46] to obtain individual variation results. The gVCF files for each sample were merged. The following conditional parameters were applied: QD 200.0 || MQ < 40.0 || MQRankSum < −12.5 || ReadPosRankSum < −8. The identified SNPs and INDELs were visualized using the CMplot package v.4.5.1 [47].

### 4.3. Linkage Disequilibrium (LD)

Linkage disequilibrium (LD) decay between male and female populations of *Procambarus clarkii* was calculated using PopLDdecay, based on squared allele frequency correlation (R^2^) statistics for loci with a minor allele frequency (MAF) > 0.01. The maximum distance between two SNPs was set at 5 kb. The Perl script Plot_MultPop.pl within PopLDdecay was utilized to calculate and visualize LD decay [48].

### 4.4. Genome-Wide Selective Sweep Analysis

Genome-wide selective sweeps across the genome were identified using fixation index (FST), nucleotide diversity (Pi), and cross-population composite likelihood ratio test (XP-CLR) methods to search for signals of differentiation between female and male populations. Pi represents the expected heterozygosity at each locus, calculated as the average number of sequence differences in a group of samples. FST, the inbreeding coefficient of the subpopulation, is used to estimate pairwise genomic differentiation between subpopulations [49]. XP-CLR is a method employed to investigate differences in the frequency of multiple alleles within a population. The Pi, FST, and XP-CLR values were sorted in descending order, and the top 1% of windows was considered as candidate regions in the studied male and female populations. The corresponding gene intervals were subsequently screened. The windows identified by these three methods are typically regarded as candidate selective sweeps. Genes showing cross-overlaps in these regions are selected as candidate genes.

### 4.5. Genome-Wide Association Study (GWAS)

Genome-wide association analysis was conducted using the Mixed Linear Model (MLM) in GEMMA software (version 0.98.5) [50]. Quantile–quantile (Q–Q) plots and Manhattan plots of SNPs, based on calculated log_10_(*p*-values), were generated from filtered SNPs and InDels.

In this study, we used the phenotypic characteristics of males and females to identify genes located in sex-determining regions. To ensure the reliability of our optimal genome-wide association study (GWAS) model analysis, we first confirmed that the genome inflation factor (λ) value close to 1.00. Q–Q plots and Manhattan plots of significant SNPs or InDels were generated using CMplot. The thresholds for genome-wide significance association were set to 0.01/N and 0.05/N, and the suggestive significance threshold was set to be 1/N, where N represents the number of SNPs and InDels used in the GWAS analysis. These thresholds were determined using the Bonferroni adjustment. LD Block Show software (v 1.4) was used to identify LD blocks of candidate gene regions.

### 4.6. Validation PCR Amplification of Male Chromosome-Specific Fragments

The SNPs and Indels in the BAM files with deduplicated were visualized using IGV (Integrative Genomics Viewer) v2.16.2 [51] to compare the differences between the two populations. The identified Indel fragments were used to develop sex-specific markers for *Procambarus clarkii*. Sex-specific markers were designed using Premier 6.0 software [52]. A total of 100 male and female *Procambarus clarkii* samples were analyzed using PCR and 2% agarose gel electrophoresis (Figure S1, Supplementary Material S5).

### 4.7. Functional Annotation of Candidate Genes for Sexdetermination

Based on the gene annotation information derived from the selected sex candidate regions, a total of five genes meet the criteria specified in the gff file. To investigate linkage disequilibrium (LD) across loci in sex-determining regions, LD Block Show v1.4 [53] was used to assess nonrandom associations. The three candidate genes were mapped at different sites in the genome. The protein sequences were extracted from the genomic coding DNA sequence (CDS) files corresponding to the three genes. These genes were associated with nonredundant protein sequences (NRs) v0.8.22.84 using diamonds [54]. The three protein sequences were extracted and converted to gene symbols using eggNOG-mapperb and UniProt online annotation tools. An online gprofiler tool [55] were converted to Entrez gene IDs. To explore the corresponding biological functions and related pathways of these candidate genes, KEGG and GO enrichment analyses were conducted using Goatools v1.4.12 and KOBAS 3.0.

### 4.8. RNA-Seq Analysis

Total RNA was extracted from the collected muscle using the Invitrogen Trizol kit (Thermo Fisher Scientific, Waltham, MA, USA) and detected by 1% agarose gel electrophoresis. RNA purity, concentration, and integrity were assessed using a NanoDrop 2000 spectrophotometer (Thermo Fisher Scientific, USA) and an Agilent 2100 Bioanalyzer (Agilent, Santa Clara, CA, USA). Illumina library construction was conducted by Biomarker Technologies (Beijing, China). Raw data quality control was performed using fastp. Quality-controlled sequences were aligned to reference genomes using Hisat2 to generate alignment data. Read count data were normalized using TMM, and differential expression analysis was conducted using DEGseq. FPKM values were calculated using StringTie software v2.2.3 [56]. Differentially expressed genes (DEGs) were identified using DESeq2 software v1.20.0 [57]. Differentially expressed genes were identified using the criteria: q < 0.05 and |Fold Change| > 2. Gene Ontology (GO) and Kyoto Encyclopedia of Genes and Genomes (KEGG) enrichment analyses were performed to determine DEG functions and associated pathways (q < 0.05).

### 4.9. Validation of Candidate Genes by RT-qPCR

Total RNA was extracted from ovary and testis tissues of *Procambarus clarkii* according to the manufacturer’s instructions of the RNA Extraction Kit (Takara, Japan). The extracted RNA was reverse-transcribed into cDNA using reagents from Thermo Fisher (China). RT-qPCR primers for these genes were designed using Primer Premier v6.0 software and are listed in Table S5. The 20 μL reaction mixture contained 1 μL cDNA, 10 μL 2× SybrGreen qPCR Master Mix, 0.4 μL each of forward and reverse primers, and 8.2 μL RNase-free water. The qPCR program consisted of an initial denaturation at 95 °C for 30 s, followed by 40 cycles of 95 °C for 5 s, 60 °C for 30 s, and 72 °C for 30 s. The primers for internal reference genes are listed in Supplementary Material S6. The relative expression levels were validated using the 2^−ΔΔCt^ method.

## Figures and Tables

**Table 1:** Statistical results of quality control information of sequencing data.

Samples	Rawreads	Rawbases	Cleanreads	Cleanbases	Cleanrate	CleanQ20	CleanQ30	Depth	GC
GTC-1	193,152,830	28,972,924,500	186,218,432	27,535,480,258	95.04%	98.85%	96.41%	10.07	43.85%
GTC-2	179,197,382	26,879,607,300	170,202,402	25,055,113,518	93.21%	98.94%	96.69%	9.16	43.75%
GTC-3	199,069,488	29,860,423,200	191,042,044	28,206,309,962	94.46%	98.88%	96.53%	10.31	43.78%
GTD-1	204,217,290	30,632,593,500	197,851,494	29,284,902,859	95.60%	98.03%	95.31%	10.71	43.25%
GTD-2	207,191,432	31,078,714,800	199,160,526	29,396,689,564	94.59%	98.86%	96.43%	10.75	43.80%
GTD-3	207,876,526	31,181,478,900	199,619,274	29,481,122,292	94.55%	97.94%	95.07%	10.78	43.36%
MTC-1	204,264,918	30,639,737,700	196,684,036	29,067,170,758	94.87%	97.97%	95.09%	10.63	43.17%
MTC-2	209,304,784	31,395,717,600	197,546,716	29,030,992,365	92.47%	98.05%	95.34%	10.61	43.34%
MTC-3	199,314,512	29,897,176,800	193,014,748	28,582,595,654	95.60%	97.98%	95.17%	10.45	43.00%
MTD-1	199,842,842	29,976,426,300	193,234,426	28,637,856,155	95.53%	97.94%	95.02%	10.47	43.32%
MTD-2	190,860,038	28,629,005,700	181,756,682	26,747,994,213	93.43%	98.91%	96.61%	9.78	43.89%
MTD-3	176,952,476	26,542,871,400	167,846,118	24,758,608,426	93.28%	98.84%	96.42%	9.05	43.91%

**Table 2:** Statistics of quality-controlled data alignment to the reference genome.

Samples	TotalReads	TotalBases	MappedReads	MappedBases	MappedReadsRate	MapDepth	Cov1X	Cov3X
GTC-1	186,218,432	27,535,480,258	184,092,905	27,218,647,300	0.9886	9.83	0.8709	0.7751
GTC-2	170,202,402	25,055,113,518	168,392,658	24,785,923,669	0.9894	8.95	0.8665	0.7631
GTC-3	191,042,044	28,206,309,962	188,921,743	27,890,640,114	0.9889	10.07	0.8747	0.7851
GTD-1	197,851,494	29,284,902,859	195,729,280	28,968,754,192	0.9893	10.46	0.8745	0.7873
GTD-2	199,160,526	29,396,689,564	197,025,587	29,078,708,101	0.9893	10.5	0.8773	0.7911
GTD-3	199,619,274	29,481,122,292	197,353,506	29,143,704,531	0.9886	10.52	0.8744	0.7887
MTC-1	196,684,036	29,067,170,758	194,515,299	28,744,047,991	0.989	10.38	0.8734	0.7868
MTC-2	197,546,716	29,030,992,365	195,583,429	28,739,457,302	0.9901	10.38	0.8721	0.7855
MTC-3	193,014,748	28,582,595,654	191,074,431	28,293,389,832	0.9899	10.22	0.869	0.7794
MTD-1	193,234,426	28,637,856,155	191,005,460	28,305,426,423	0.9885	10.22	0.8742	0.7849
MTD-2	181,756,682	26,747,994,213	179,857,259	26,465,480,807	0.9895	9.56	0.8732	0.7773
MTD-3	167,846,118	24,758,608,426	166,078,347	24,495,385,469	0.9895	8.84	0.8631	0.7563

## Data Availability

The data from the study are available from the corresponding authors upon reasonable request.

## Supplementary Materials

The following supporting information can be downloaded at: https://www.mdpi.com/article/10.3390/ijms27020938/s1.
