# Supplementary material for: Whole-Genome Resequencing Analysis Reveals Insights into Sex Determination and Gene Loci Associated with Sex Differences in Procambarus clarkii
Source: Int J Mol Sci. 2026 Jan 17;27(2):938. doi: 10.3390/ijms27020938 (PMC12842422; doi:10.3390/ijms27020938)
Supplement: Supplementary file 1 [file ijms-27-00938-s001.zip › Supplementary Material S1.pdf]

Table S1. Statistical results of quality control information of sequencing data from 89 samples

Samples	Rawreads	Rawbases	Cleanreads	Cleanbases	Cleanrate	CleanQ20	CleanQ30	Depth	GC_rate
GTC-1	193,152,830	28,972,924,500	186,218,432	27,535,480,258	95.04%	98.85%	96.41%	10.07	43.85%
GTC-2	179,197,382	26,879,607,300	170,202,402	25,055,113,518	93.21%	98.94%	96.69%	9.16	43.75%
GTC-3	199,069,488	29,860,423,200	191,042,044	28,206,309,962	94.46%	98.88%	96.53%	10.31	43.78%
GTD-1	204,217,290	30,632,593,500	197,851,494	29,284,902,859	95.60%	98.03%	95.31%	10.71	43.25%
GTD-2	207,191,432	31,078,714,800	199,160,526	29,396,689,564	94.59%	98.86%	96.43%	10.75	43.80%
GTD-3	207,876,526	31,181,478,900	199,619,274	29,481,122,292	94.55%	97.94%	95.07%	10.78	43.36%
MTC-1	204,264,918	30,639,737,700	196,684,036	29,067,170,758	94.87%	97.97%	95.09%	10.63	43.17%
MTC-2	209,304,784	31,395,717,600	197,546,716	29,030,992,365	92.47%	98.05%	95.34%	10.61	43.34%
MTC-3	199,314,512	29,897,176,800	193,014,748	28,582,595,654	95.60%	97.98%	95.17%	10.45	43.00%
MTD-1	199,842,842	29,976,426,300	193,234,426	28,637,856,155	95.53%	97.94%	95.02%	10.47	43.32%
MTD-2	190,860,038	28,629,005,700	181,756,682	26,747,994,213	93.43%	98.91%	96.61%	9.78	43.89%
MTD-3	176,952,476	26,542,871,400	167,846,118	24,758,608,426	93.28%	98.84%	96.42%	9.05	43.91%
F-L-HA-01	193,227,610	28,984,141,500	186,958,046	27,618,435,902	95.29%	98.81%	95.91%	10.10	43.92%
F-L-HA-02	183,385,078	27,507,761,700	176,488,790	25,950,026,840	94.34%	98.88%	96.15%	9.49	43.80%
F-L-HA-03	191,652,836	28,747,925,400	185,248,544	27,326,723,364	95.06%	98.77%	95.76%	9.99	43.29%
F-L-HA-04	178,016,416	26,702,462,400	171,650,952	25,314,695,431	94.80%	98.85%	96.04%	9.25	43.22%
F-L-HA-05	231,412,762	34,711,914,300	223,951,808	33,086,241,301	95.32%	98.82%	95.91%	12.10	43.36%
F-L-MAS-01	199,970,570	29,995,585,500	192,426,730	28,354,914,236	94.53%	98.98%	96.71%	10.37	43.28%
F-L-MAS-02	203,206,732	30,481,009,800	195,124,786	28,739,811,006	94.29%	98.75%	96.28%	10.51	42.73%
F-L-MAS-03	202,552,176	30,382,826,400	195,512,550	28,800,020,570	94.79%	98.55%	95.69%	10.53	44.87%
F-L-MAS-04	630,484,940	94,572,741,000	606,133,698	89,277,789,204	94.40%	99.00%	96.75%	32.64	43.04%
F-L-MAS-05	202,188,842	30,328,326,300	194,407,702	28,632,854,745	94.41%	99.05%	96.92%	10.47	43.29%
F-L-WH-01	201,331,040	30,199,656,000	194,625,466	28,763,666,297	95.25%	99.03%	96.86%	10.52	42.77%
F-L-WH-02	203,564,724	30,534,708,600	196,028,488	28,885,679,793	94.60%	98.81%	96.44%	10.56	43.81%
F-L-WH-03	219,018,534	32,852,780,100	210,592,032	31,052,668,358	94.52%	98.98%	96.68%	11.35	42.74%
F-L-WH-04	183,321,624	27,498,243,600	176,762,010	26,077,137,323	94.83%	98.99%	96.73%	9.53	42.63%
F-L-YY-01	210,405,032	31,560,754,800	203,813,348	30,106,869,995	95.39%	98.82%	95.93%	11.01	43.54%
F-L-YY-02	194,045,612	29,106,841,800	186,614,592	27,449,968,980	94.31%	98.81%	95.90%	10.04	44.45%
F-L-YY-03	242,250,102	36,337,515,300	235,243,940	34,779,226,587	95.71%	98.74%	95.65%	12.71	43.54%
F-L-YY-04	186,249,332	27,937,399,800	180,878,732	26,752,868,907	95.76%	98.83%	95.95%	9.78	43.21%
F-L-YY-05	250,515,372	37,577,305,800	242,320,820	35,714,819,784	95.04%	98.84%	96.00%	13.06	43.51%
F-S-HA-01	197,468,920	29,620,338,000	191,337,020	28,268,154,696	95.43%	98.78%	95.80%	10.33	43.32%
F-S-HA-02	238,778,252	35,816,737,800	231,557,150	34,203,210,861	95.50%	98.84%	95.98%	12.50	43.19%
F-S-HA-03	203,044,520	30,456,678,000	196,857,596	29,083,816,021	95.49%	98.56%	95.54%	10.63	42.78%
F-S-HA-04	235,995,722	35,399,358,300	228,276,922	33,663,268,556	95.10%	98.83%	95.97%	12.31	42.81%
F-S-HA-05	247,199,418	37,079,912,700	242,406,832	36,103,779,578	97.37%	98.59%	95.66%	13.20	42.48%
F-S-MAS-01	211,482,808	31,722,421,200	203,339,260	29,955,939,887	94.43%	98.84%	96.53%	10.95	42.71%
F-S-MAS-02	207,650,816	31,147,622,400	199,566,692	29,419,241,831	94.45%	98.99%	96.76%	10.76	42.86%
F-S-MAS-03	216,713,554	32,507,033,100	209,389,948	30,885,914,534	95.01%	98.81%	96.42%	11.29	42.87%
F-S-MAS-04	201,082,718	30,162,407,700	193,410,552	28,488,685,172	94.45%	98.99%	96.72%	10.41	43.11%
F-S-MAS-05	200,046,988	30,007,048,200	192,893,624	28,459,899,029	94.84%	98.77%	96.30%	10.40	43.26%
F-S-WH-01	176,691,808	26,503,771,200	169,773,198	25,056,910,710	94.54%	98.99%	96.75%	9.16	42.98%

F-S-WH-02	224,072,356	33,610,853,400	215,740,370	31,814,957,109	94.66%	98.82%	96.46%	11.63	42.51%
F-S-WH-03	203,649,844	30,547,476,600	196,426,368	28,976,457,671	94.86%	98.82%	96.27%	10.59	42.38%
F-S-WH-04	182,536,724	27,380,508,600	175,251,690	25,779,747,608	94.15%	99.04%	96.92%	9.42	42.62%
F-S-YY-01	203,756,804	30,563,520,600	196,684,076	29,002,723,867	94.89%	98.77%	96.17%	10.60	43.26%
F-S-YY-02	206,018,762	30,902,814,300	199,646,684	29,519,527,374	95.52%	98.40%	95.09%	10.79	42.52%
F-S-YY-03	208,628,060	31,294,209,000	201,604,388	29,778,127,153	95.16%	98.39%	95.03%	10.89	42.74%
F-S-YY-04	217,189,524	32,578,428,600	211,032,370	31,214,369,826	95.81%	98.83%	95.94%	11.41	43.07%
F-S-YY-05	202,162,856	30,324,428,400	195,112,536	28,780,951,385	94.91%	98.79%	95.81%	10.52	42.59%
M-L-HA-01	209,183,480	31,377,522,000	203,438,410	30,119,234,987	95.99%	98.81%	95.87%	11.01	43.50%
M-L-HA-02	188,012,408	28,201,861,200	183,048,314	27,085,282,149	96.04%	98.73%	95.60%	9.90	43.56%
M-L-HA-03	213,916,552	32,087,482,800	207,420,600	30,616,118,300	95.41%	98.77%	95.75%	11.19	43.71%
M-L-HA-04	180,977,438	27,146,615,700	174,802,378	25,771,317,650	94.93%	98.80%	95.86%	9.42	43.03%
M-L-HA-05	214,913,910	32,237,086,500	207,818,334	30,670,222,133	95.14%	98.81%	95.89%	11.21	42.83%
M-L-MAS-01	194,755,174	29,213,276,100	187,149,504	27,591,666,689	94.45%	99.02%	96.84%	10.09	42.82%
M-L-MAS-02	180,058,568	27,008,785,200	173,461,220	25,592,496,359	94.76%	98.98%	96.71%	9.36	43.04%
M-L-MAS-03	215,030,786	32,254,617,900	207,391,748	30,576,260,475	94.80%	99.01%	96.81%	11.18	43.25%
M-L-MAS-04	205,591,556	30,838,733,400	197,635,016	29,160,096,020	94.56%	98.82%	96.47%	10.66	42.87%
M-L-MAS-05	208,014,732	31,202,209,800	200,557,164	29,595,860,871	94.85%	98.71%	96.10%	10.82	43.10%
M-L-WH-01	188,149,312	28,222,396,800	180,706,696	26,651,289,762	94.43%	98.87%	96.09%	9.74	42.58%
M-L-WH-02	202,159,762	30,323,964,300	195,646,564	28,900,549,788	95.31%	98.62%	95.71%	10.57	42.58%
M-L-WH-03	203,094,092	30,464,113,800	195,864,128	28,919,260,961	94.93%	98.78%	95.78%	10.57	42.72%
M-L-WH-04	177,200,094	26,580,014,100	171,291,152	25,275,068,909	95.09%	98.78%	95.78%	9.24	42.75%
M-L-WH-05	214,838,760	32,225,814,000	207,717,998	30,698,603,882	95.26%	98.66%	95.83%	11.22	42.73%
M-L-YY-01	207,491,426	31,123,713,900	199,893,866	29,449,843,145	94.62%	98.83%	95.97%	10.77	42.95%
M-L-YY-02	176,461,366	26,469,204,900	170,400,112	25,119,071,183	94.90%	98.53%	94.95%	9.18	42.93%
M-L-YY-03	213,054,376	31,958,156,400	205,927,468	30,380,360,983	95.06%	98.78%	95.76%	11.11	43.25%
M-L-YY-04	224,698,894	33,704,834,100	216,672,030	31,875,507,985	94.57%	98.85%	96.06%	11.65	43.64%
M-L-YY-05	215,879,760	32,381,964,000	208,583,482	30,751,534,376	94.97%	98.84%	95.99%	11.24	43.79%
M-L-YY-06	202,321,908	30,348,286,200	196,852,242	29,134,605,928	96%	98.69%	95.90%	10.65	43.71%
M-S-HA-01	217,336,242	32,600,436,300	210,116,698	31,027,091,646	95.17%	98.63%	95.75%	11.34	42.59%
M-S-HA-02	195,047,290	29,257,093,500	188,860,260	27,904,707,182	95.38%	98.60%	95.16%	10.20	43.01%
M-S-HA-03	183,161,894	27,474,284,100	177,156,722	26,096,206,129	94.98%	98.80%	95.89%	9.54	43.65%
M-S-HA-04	224,418,002	33,662,700,300	217,090,580	32,032,857,975	95.16%	98.79%	95.84%	11.71	43.07%
M-S-HA-05	215,963,998	32,394,599,700	209,123,198	30,903,651,807	95.40%	98.66%	95.86%	11.30	42.73%
M-S-MAS-01	205,816,336	30,872,450,400	197,777,356	29,112,758,176	94.30%	98.81%	96.46%	10.64	43.34%
M-S-MAS-02	210,814,618	31,622,192,700	203,197,566	29,944,512,330	94.69%	98.79%	96.35%	10.95	43.47%
M-S-MAS-03	206,952,146	31,042,821,900	199,015,784	29,328,182,470	94.48%	98.81%	96.47%	10.72	42.99%
M-S-MAS-04	183,572,856	27,535,928,400	176,441,950	25,999,360,507	94.42%	99.00%	96.76%	9.50	43.10%
M-S-MAS-05	216,099,598	32,414,939,700	208,993,010	30,877,638,073	95.26%	98.77%	96.30%	11.29	42.91%
M-S-WH-01	188,080,826	28,212,123,900	181,718,126	26,848,146,999	95.17%	98.99%	96.75%	9.82	42.47%
M-S-WH-02	206,024,154	30,903,623,100	198,824,092	29,335,717,570	94.93%	98.79%	96.41%	10.72	42.48%
M-S-WH-03	193,500,408	29,025,061,200	186,907,584	27,587,468,747	95.05%	99.00%	96.79%	10.09	43.08%
M-S-WH-04	221,977,052	33,296,557,800	213,260,474	31,420,213,878	94.36%	98.82%	96.47%	11.49	42.77%
M-S-YY-01	231,812,812	34,771,921,800	223,266,608	32,864,167,713	94.51%	98.87%	96.09%	12.01	43.50%

M-S-YY-02	214,591,788	32,188,768,200	206,927,004	30,482,049,647	94.70%	98.67%	95.89%	11.14	43.53%
M-S-YY-03	182,228,878	27,334,331,700	176,721,468	26,101,704,950	95.49%	98.80%	95.84%	9.54	43.62%
M-S-YY-04	182,819,846	27,422,976,900	176,835,918	26,120,575,865	95.25%	98.75%	95.67%	9.55	42.94%
