# Supplementary material for: Whole-Genome Resequencing Analysis Reveals Insights into Sex Determination and Gene Loci Associated with Sex Differences in Procambarus clarkii
Source: Int J Mol Sci. 2026 Jan 17;27(2):938. doi: 10.3390/ijms27020938 (PMC12842422; doi:10.3390/ijms27020938)
Supplement: Supplementary file 1 [file ijms-27-00938-s001.zip › Supplementary Material S2.pdf]

Table S2. Statistics of quality-controlled data alignment to the reference genome

Sample	TotalReads	TotalBases	MappedReads	MappedBases	MappedReadsRate	MapDepth	Cov1X	Cov3X
GTC-1	186,218,432	27,535,480,258	184,092,905	27,218,647,300	0.9886	9.83	0.8709	0.7751
GTC-2	170,202,402	25,055,113,518	168,392,658	24,785,923,669	0.9894	8.95	0.8665	0.7631
GTC-3	191,042,044	28,206,309,962	188,921,743	27,890,640,114	0.9889	10.07	0.8747	0.7851
GTD-1	197,851,494	29,284,902,859	195,729,280	28,968,754,192	0.9893	10.46	0.8745	0.7873
GTD-2	199,160,526	29,396,689,564	197,025,587	29,078,708,101	0.9893	10.5	0.8773	0.7911
GTD-3	199,619,274	29,481,122,292	197,353,506	29,143,704,531	0.9886	10.52	0.8744	0.7887
MTC-1	196,684,036	29,067,170,758	194,515,299	28,744,047,991	0.989	10.38	0.8734	0.7868
MTC-2	197,546,716	29,030,992,365	195,583,429	28,739,457,302	0.9901	10.38	0.8721	0.7855
MTC-3	193,014,748	28,582,595,654	191,074,431	28,293,389,832	0.9899	10.22	0.869	0.7794
MTD-1	193,234,426	28,637,856,155	191,005,460	28,305,426,423	0.9885	10.22	0.8742	0.7849
MTD-2	181,756,682	26,747,994,213	179,857,259	26,465,480,807	0.9895	9.56	0.8732	0.7773
MTD-3	167,846,118	24,758,608,426	166,078,347	24,495,385,469	0.9895	8.84	0.8631	0.7563
F-L-HA-01	186,167,511	27,501,564,376	184,173,317	27,204,577,665	0.9893	9.82	0.8616	0.7571
F-L-HA-02	175,755,450	25,842,075,310	173,881,632	25,563,681,597	0.9893	9.23	0.8611	0.7521
F-L-HA-03	184,432,220	27,206,167,053	182,408,845	26,904,915,268	0.989	9.71	0.8688	0.7685
F-L-HA-04	170,822,644	25,192,405,650	168,124,636	24,790,867,154	0.9842	8.95	0.8626	0.7522
F-L-HA-05	222,734,924	32,906,198,133	219,994,491	32,497,956,832	0.9877	11.73	0.879	0.7949
F-L-MAS-01	191,251,513	28,181,459,455	188,269,473	27,738,070,393	0.9844	10.02	0.8725	0.7735
F-L-MAS-02	193,861,432	28,553,353,341	190,847,916	28,105,404,284	0.9845	10.15	0.873	0.7739
F-L-MAS-03	194,277,631	28,618,954,982	191,509,841	28,208,214,058	0.9858	10.19	0.855	0.7391
F-L-MAS-04	598,974,337	88,219,851,645	591,590,646	87,122,065,450	0.9877	31.46	0.9221	0.8816
F-L-MAS-05	193,367,346	28,479,609,228	189,706,019	27,935,370,474	0.9811	10.09	0.8747	0.7745
F-L-WH-01	193,558,806	28,605,941,459	191,467,321	28,294,553,979	0.9892	10.22	0.8723	0.7766
F-L-WH-02	195,101,290	28,749,059,206	193,032,112	28,441,454,871	0.9894	10.27	0.8702	0.7696
F-L-WH-03	209,187,554	30,844,944,679	206,862,137	30,499,114,841	0.9889	11.01	0.8816	0.7926
F-L-WH-04	175,863,405	25,944,305,845	173,842,773	25,643,611,452	0.9885	9.26	0.8715	0.7641
F-L-YY-01	201,389,976	29,748,630,379	181,887,131	26,837,517,167	0.9032	9.69	0.8671	0.7592
F-L-YY-02	185,799,895	27,330,177,667	183,467,250	26,983,707,937	0.9874	9.74	0.8621	0.7547
F-L-YY-03	233,946,401	34,586,996,641	231,232,050	34,182,502,204	0.9884	12.34	0.8783	0.7995
F-L-YY-04	180,046,412	26,629,575,072	177,823,049	26,298,278,545	0.9877	9.5	0.8669	0.7654
F-L-YY-05	240,684,540	35,472,845,065	236,913,897	34,912,228,936	0.9843	12.61	0.8816	0.8022
F-S-HA-01	190,537,405	28,149,707,972	188,300,581	27,816,475,220	0.9883	10.04	0.8676	0.7687
F-S-HA-02	230,208,113	34,003,763,885	227,308,670	33,571,930,433	0.9874	12.12	0.8795	0.7966
F-S-HA-03	195,861,003	28,936,401,731	192,851,871	28,488,073,900	0.9846	10.29	0.8685	0.7731
F-S-HA-04	227,113,702	33,491,687,526	224,048,167	33,035,133,843	0.9865	11.93	0.8793	0.7982
F-S-HA-05	240,793,469	35,863,662,682	235,970,113	35,142,170,883	0.98	12.69	0.8838	0.8039
F-S-MAS-01	202,142,727	29,779,453,782	199,995,524	29,460,107,749	0.9894	10.64	0.8758	0.7827
F-S-MAS-02	198,245,010	29,224,142,048	195,777,074	28,857,090,957	0.9876	10.42	0.8767	0.7825
F-S-MAS-03	208,322,353	30,728,118,125	205,637,418	30,328,797,949	0.9871	10.95	0.877	0.7876
F-S-MAS-04	192,587,506	28,367,694,656	189,645,046	27,930,315,247	0.9847	10.08	0.876	0.7793
F-S-MAS-05	192,090,306	28,341,633,843	189,690,941	27,984,563,366	0.9875	10.1	0.8726	0.7741
F-S-WH-01	168,838,157	24,918,648,521	166,931,671	24,634,895,858	0.9887	8.89	0.8631	0.7525

F-S-WH-02	214,324,650	31,605,912,400	211,874,667	31,241,495,699	0.9886	11.28	0.8798	0.7934
F-S-WH-03	195,108,640	28,781,917,156	192,965,250	28,463,108,274	0.989	10.28	0.87	0.7731
F-S-WH-04	174,261,422	25,633,590,273	172,297,227	25,341,666,027	0.9887	9.15	0.8667	0.7578
F-S-YY-01	195,452,079	28,820,614,601	192,973,977	28,452,183,079	0.9873	10.27	0.8703	0.7761
F-S-YY-02	198,349,216	29,327,300,335	195,736,849	28,938,343,777	0.9868	10.45	0.868	0.7746
F-S-YY-03	200,347,251	29,591,885,158	196,915,228	29,080,401,914	0.9829	10.5	0.8713	0.7776
F-S-YY-04	209,819,154	31,034,480,688	207,113,576	30,631,246,828	0.9871	11.06	0.8725	0.7836
F-S-YY-05	194,065,343	28,626,152,427	191,976,789	28,315,189,215	0.9892	10.22	0.8717	0.7776
M-L-HA-01	202,423,068	29,968,830,773	199,781,188	29,575,019,820	0.9869	10.68	0.8707	0.7768
M-L-HA-02	182,293,308	26,973,378,170	180,158,644	26,655,092,186	0.9883	9.62	0.8645	0.7597
M-L-HA-03	206,475,508	30,476,274,992	204,004,760	30,108,354,538	0.988	10.87	0.8748	0.7824
M-L-HA-04	174,067,618	25,662,758,313	172,141,521	25,376,047,890	0.9889	9.16	0.8631	0.7578
M-L-HA-05	206,835,398	30,525,071,339	204,309,356	30,148,751,521	0.9878	10.89	0.8793	0.7909
M-L-MAS-01	186,354,728	27,474,479,428	182,110,375	26,843,218,050	0.9772	9.69	0.8769	0.7739
M-L-MAS-02	172,648,153	25,472,634,503	170,533,190	25,157,865,993	0.9877	9.08	0.8678	0.7578
M-L-MAS-03	206,303,053	30,415,818,372	201,761,576	29,740,503,588	0.978	10.74	0.8742	0.7821
M-L-MAS-04	196,588,509	29,005,716,876	193,973,660	28,616,675,156	0.9867	10.33	0.8749	0.7807
M-L-MAS-05	199,487,181	29,437,928,548	196,466,185	28,988,492,996	0.9849	10.47	0.8745	0.78
M-L-WH-01	179,822,426	26,520,563,164	177,934,451	26,239,542,364	0.9895	9.47	0.8668	0.7635
M-L-WH-02	194,590,170	28,744,355,590	192,556,237	28,441,241,437	0.9895	10.27	0.8725	0.779
M-L-WH-03	194,811,821	28,763,815,887	192,771,091	28,459,747,181	0.9895	10.28	0.8709	0.7775
M-L-WH-04	170,452,015	25,150,818,512	168,570,956	24,870,758,537	0.989	8.98	0.8622	0.753
M-L-WH-05	206,374,602	30,499,689,900	204,216,950	30,178,186,352	0.9895	10.9	0.8724	0.7835
M-L-YY-01	198,846,232	29,295,109,284	196,456,244	28,939,582,664	0.988	10.45	0.873	0.781
M-L-YY-02	169,612,781	25,002,838,481	167,589,404	24,701,891,979	0.9881	8.92	0.8565	0.7472
M-L-YY-03	204,932,255	30,233,344,523	202,638,818	29,891,876,158	0.9888	10.79	0.8756	0.7866
M-L-YY-04	215,518,167	31,705,464,437	212,833,739	31,306,668,401	0.9875	11.3	0.8743	0.787
M-L-YY-05	207,354,758	30,569,976,174	204,016,283	30,074,431,882	0.9839	10.86	0.8709	0.7789
M-L-YY-06	195,589,349	28,947,332,788	192,008,353	28,413,944,416	0.9817	10.26	0.8697	0.7711
M-S-HA-01	209,038,663	30,867,645,455	206,708,997	30,520,639,114	0.9889	11.02	0.8743	0.787
M-S-HA-02	187,972,947	27,773,703,647	185,645,501	27,426,887,125	0.9876	9.9	0.8682	0.77
M-S-HA-03	176,143,719	25,946,906,446	174,370,670	25,683,132,139	0.9899	9.27	0.8606	0.7531
M-S-HA-04	215,780,059	31,839,114,781	213,189,646	31,453,296,391	0.988	11.36	0.8752	0.7895
M-S-HA-05	207,869,191	30,717,836,131	205,341,667	30,341,109,301	0.9878	10.96	0.8711	0.781
M-S-MAS-01	196,840,573	28,975,140,267	194,260,637	28,591,816,685	0.9869	10.32	0.8751	0.7794
M-S-MAS-02	202,319,008	29,815,118,480	199,839,594	29,446,500,442	0.9877	10.63	0.8742	0.7801
M-S-MAS-03	198,006,762	29,179,432,788	195,165,107	28,757,168,139	0.9856	10.38	0.8749	0.7791
M-S-MAS-04	175,301,341	25,831,059,441	173,259,812	25,527,361,077	0.9884	9.22	0.8713	0.7629
M-S-MAS-05	207,706,167	30,687,459,823	204,561,182	30,219,365,786	0.9849	10.91	0.8774	0.7871
M-S-WH-01	180,499,177	26,667,756,218	178,454,319	26,363,091,537	0.9887	9.52	0.8696	0.765
M-S-WH-02	197,613,617	29,156,473,101	195,232,190	28,802,163,640	0.9879	10.4	0.8747	0.7797
M-S-WH-03	185,906,137	27,439,754,350	183,853,504	27,134,230,644	0.989	9.8	0.8694	0.7684
M-S-WH-04	211,704,503	31,190,436,769	209,169,435	30,813,753,751	0.988	11.13	0.8782	0.7896
M-S-YY-01	222,017,259	32,679,966,245	219,095,204	32,245,542,646	0.9868	11.64	0.8767	0.7923

M-S-YY-02	205,636,505	30,291,580,467	203,213,814	29,931,314,474	0.9882	10.81	0.8703	0.7767
M-S-YY-03	175,932,679	25,985,354,016	173,775,237	25,664,097,772	0.9877	9.27	0.8634	0.7571
M-S-YY-04	175,964,820	25,992,000,315	173,546,862	25,631,959,110	0.9863	9.25	0.8615	0.7572
