# Supplementary material for: Whole-Genome Resequencing Analysis Reveals Insights into Sex Determination and Gene Loci Associated with Sex Differences in Procambarus clarkii
Source: Int J Mol Sci. 2026 Jan 17;27(2):938. doi: 10.3390/ijms27020938 (PMC12842422; doi:10.3390/ijms27020938)
Supplement: Supplementary file 1 [file ijms-27-00938-s001.zip › Supplementary Material S3.pdf]

Table S3. The results of Gene Ontology (GO) enrichment analysis for candidate genes

GO ID	Go terms	GeneRatio	BgRatio	Pvalue	Qvalue	Count
GO:0005886	plasma membrane	1/5	3129/37933	0.653	1	1
GO:0016020	membrane	1/5	6664/37933	0.903	1	1
GO:0055085	transmembrane transport	1/5	737/37933	0.40224	1	1
GO:0015293	symporter activity	1/5	175/37933	0.1062	1	1
GO:0071704	organic substance metabolic process	1/5	7493/37933	0.3883	1	1
GO:0046872	metal ion binding	1/5	3519/37933	0.0439	1	1
GO:0005524	ATP binding	1/5	1271/37933	0.5743	1	1
GO:0005829	cytosol	1/5	2181/37933	0.795	1	1
GO:0005634	nucleus	1/5	4661/37933	0.872	1	1
