# Supplementary material for: Whole-Genome Resequencing Analysis Reveals Insights into Sex Determination and Gene Loci Associated with Sex Differences in Procambarus clarkii
Source: Int J Mol Sci. 2026 Jan 17;27(2):938. doi: 10.3390/ijms27020938 (PMC12842422; doi:10.3390/ijms27020938)
Supplement: Supplementary file 1 [file ijms-27-00938-s001.zip › Supplementary Material S4.pdf]

Table S4. The results of KEGG enrichment analysis for candidate genes

ID	Description	GeneRatio	BgRatio	Pvalue	Qvalue	Count
ko04064	NF-kappa B signaling pathway	1/4	52/4463	0.438642662	0.534250003	1
ko04921	Oxytocin signaling pathway	1/4	107/4463	0.111927206	0.456545073	1
ko04723	Retrograde endocannabinoid signaling	1/4	41/4463	0.365353489	0.496106323	1
ko04370	VEGF signaling pathway	1/4	30/4463	0.282715805	0.496106323	1
ko04726	Serotonergic synapse	1/4	55/4463	0.457147434	0.540557558	1
ko00590	Arachidonic acid metabolism	1/4	40/4463	0.358243823	0.496106323	1
ko04923	Regulation of lipolysis in adipocyte	1/4	37/4463	0.336443446	0.496106323	1
ko00970	Aminoacyl-tRNA biosynthesis	1/4	43/4463	0.426196385	0.912865733	1
ko03460	Fanconi anemia pathway	1/4	43/4463	0.426196385	0.912865733	1
ko04976	Bile secretion	1/4	42/4463	0.931147867	0.912865733	1
