# Supplementary material for: Whole-Genome Resequencing Analysis Reveals Insights into Sex Determination and Gene Loci Associated with Sex Differences in Procambarus clarkii
Source: Int J Mol Sci. 2026 Jan 17;27(2):938. doi: 10.3390/ijms27020938 (PMC12842422; doi:10.3390/ijms27020938)

Figure S1 shows the results of PCR-based genetic sex identification in Procambarus clarkii, as revealed by agarose gel electrophoresis.

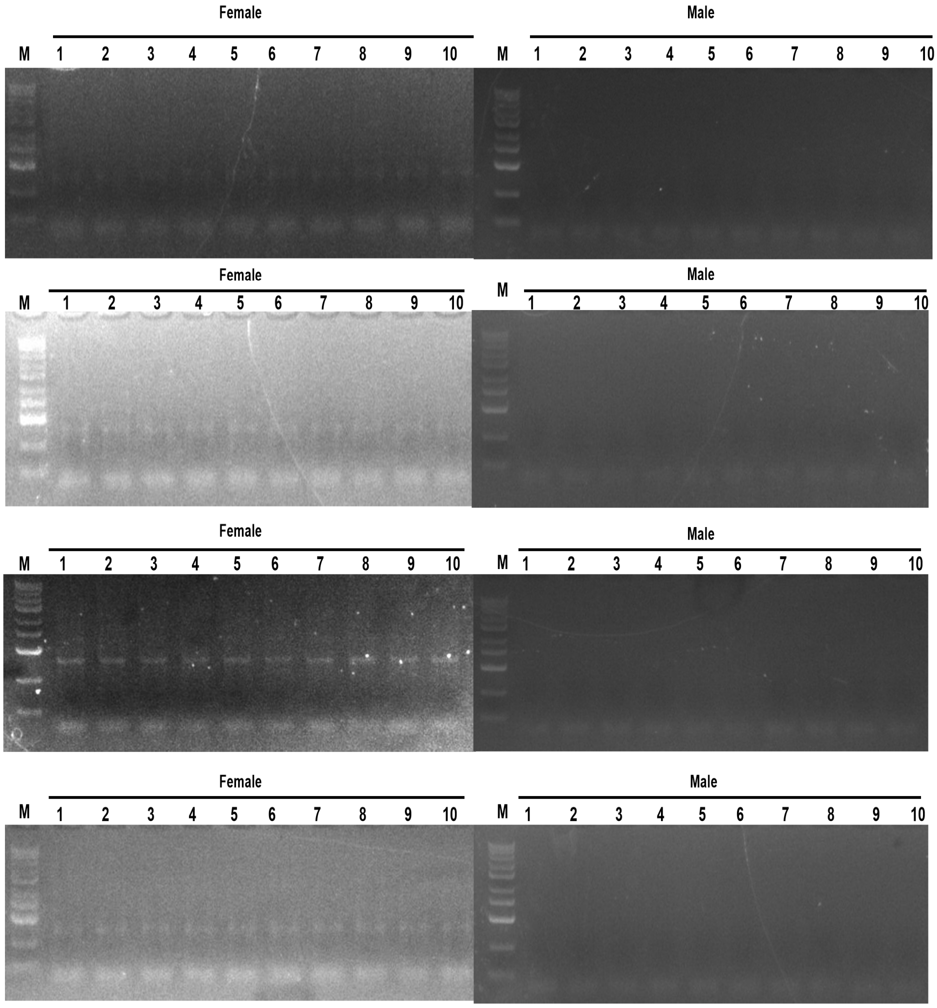

Supplement: Supplementary file 1 [file ijms-27-00938-s001.zip › Supplementary Material S5.pdf]
