# Supplementary material for: Whole-Genome Resequencing Analysis Reveals Insights into Sex Determination and Gene Loci Associated with Sex Differences in Procambarus clarkii
Source: Int J Mol Sci. 2026 Jan 17;27(2):938. doi: 10.3390/ijms27020938 (PMC12842422; doi:10.3390/ijms27020938)
Supplement: Supplementary file 1 [file ijms-27-00938-s001.zip › Supplementary Material S6.pdf]

Table S5 Primers used for the qRT-PCR analysis of differently expressed genes in the *Procambarus clarkii*

Gene name	Primers (5' → 3')	
PTGS2	Forward prime	GGCACTGAACACTATGGC
	Reverse primer	TGCTTATGAATGAATGGGAT
NPC1	Forward prime	CGAGTGAGGTCCCTGTTA
	Reverse primer	CTTTGGAAATCCCTTTAGAT
SOAT	Forward prime	ATTCTATGCCCTCCTACCC
	Reverse primer	GGTGCTGGACGTAATCCT
FANCD2	Forward prime	TGGACAAATCCCTACGCC
	Reverse primer	GCCTTGACCCGATACACC
ValRS	Forward prime	CCAAGAAAGCCGCTAAAT
	Reverse primer	CAGCTCCACGCTAACATC
TUBA2	Forward prime	CCCTCGCATCCACTTCC
	Reverse primer	CTGAATCTGTCGCTTAGTCTTTAT
GSTT4	Forward prime	GGTGGACGAATACCTGGAT
	Reverse primer	AGTAATGAATGGTTTAGAGCC
ATK	Forward prime	TCAATGGAAACCCTGTAAG
	Reverse primer	GGTCGCAGATGATGTCGT
GLIS2	Forward prime	ACAAACCCTACGAGTGCCG
	Reverse primer	GAGCGATGACCCTGAAGAAG
FLC	Forward prime	AACAATAACAACATCAGGCTAC
	Reverse primer	GACCAGGACGCTCTACAT
SLC5A10	Forward prime	GAAGCGGATGAATGAGCG
	Reverse primer	GCGAAGTACATAAAGTTGAGGC
RPII140	Forward prime	ATTCTATTCGACTTTCTCCCGTTTC
	Reverse primer	CGCCTGCTTCACTGTCCTCA
GLS	Forward prime	AGGACGACACGGTTACGC
	Reverse primer	GCAGATGAGTCTTTAGGGTTA
18S rRNA	Forward prime	GTCAGGTCATCACCATCGGCA
	Reverse primer	CGGTCTCGTGAACACCAGCA
